# Activation of Specific Reagents in Molecular Films by Sub-Ionization Electrons: Chlorobenzene/Water Films

**DOI:** 10.3390/ijms26178751

**Published:** 2025-09-08

**Authors:** Hassan Abdoul-Carime, Janina Kopyra

**Affiliations:** 1 Institut de Physique des 2 Infinis, Universite Claude Bernard Lyon 1, CNRS/IN2P3, UMR5822, F-69622 Lyon, France; 2 Faculty of Sciences, Siedlce University, 3 Maja 54, 08-110 Siedlce, Poland; janina.kopyra@uws.edu.pl

**Keywords:** bi-reactant molecular film, low-energy electron, controlled reaction, specific-molecule activation, chlorobenzene/water film

## Abstract

Control over chemical reactivity remains a fundamental challenge in synthesis chemistry, where targeting a specific reactant represents the ultimate goal. While photoactivation is a well-established approach for selective excitation, electron-induced chemistry offers a complementary pathway with high efficacy. In this study, we investigate the effects of low-energy electron irradiation on prototypical chlorobenzene/water molecular films, demonstrating that chlorobenzene can be selectively dissociated via a resonant process occurring at ~1 eV. At higher electron energies (>6 eV), multiple reaction pathways become accessible, including the fragmentation of both water and chlorobenzene molecules. Our study provides a perspective strategy for achieving reagent-specific control in complex molecular assemblies via low-energy electrons, offering new insights into electron-driven surface chemistry and reaction dynamics at the molecular level.

## 1. Introduction

The ability to target and control the issue of chemical reactions is probably one of the most important challenges in synthesis. Such control can be achieved through a variety of strategies, including the use of an external stimulus to direct chemical reactions [1]. Photoactivation is one well-established external stimulus technique [1], in which photons of a specific wavelength trigger chemistry at the molecular level by activating specific molecules. With the development of attosecond laser sources, a new method of initiating chemical reactions by targeting the dynamics of electrons in molecules has emerged, i.e., attochemistry [2,3]. Controlling processes on the scale of a single molecule by targeting a specific reactant thus represents the ultimate limit of synthesis.

Control of chemical reactions by electron beams is another possible external stimulus for selective synthesis [4]. Low-energy (<10 eV) electron beam and scanning tunneling microscopy techniques have been applied to induce reactions in homogeneous films [5] or on spatially localized single molecules on a metal surface [6,7,8]. For instance, for the electron beam methodology, it has been shown that the synthesis of specific ethylene molecules from irradiation of a film of dimethyl sulfide can be controlled by the energy of the particles [5]. This selectivity relies on the preparation of target molecules in certain specific quantum states, allowing access to particular fragmentation channels by electrons of appropriate energy—similarly to the use of photons at a given wavelength, but using electrons instead. This resonant fragmentation process induced by electrons is known as dissociative electron attachment (DEA) [9,10]. The colliding electron can either accommodate a virtually empty molecular orbital (shape resonance) or excite a core electron, being concomitantly trapped by the positive core of the molecule (core-excited resonance), forming a transitory negative anion. The latter decays through dissociation, producing a negative fragment and at least one neutral counterpart. If the neutral fragment is a reactive radical, reactions can take place in the presence of neighboring molecules.

Thus, in a medium composed of at least two different reagents, A and B (or a heterogeneous medium), randomly dispersed in space, it is then formally possible to initiate a chemical reaction by targeting only a specific quantum state of A without exciting B, by controlling only the energy of the irradiating electron beam [4].

Here we show that specific molecules can be activated in a heterogeneous medium by electrons, by studying the prototypical binary system of chlorobenzene (C_6_H_5_Cl) in water (H_2_O) ice. Indeed, at electron energies below 2 eV, only chlorobenzene is fragmented into a Cl^−^ anion and a neutral phenyl radical (C_6_H_5_) by the irradiating particles, which are responsible for subsequent reaction steps, leading to the production of phenol and benzene.

## 2. Results and Discussion

**Figure 1:**
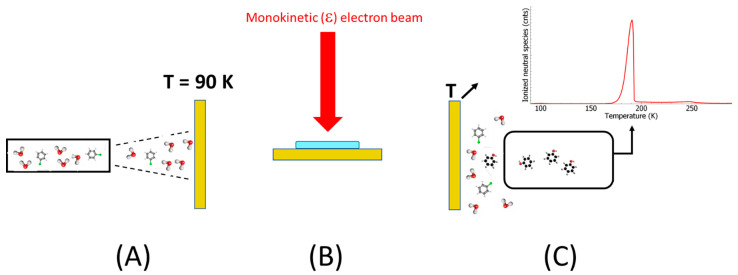
(**A**) The film is deposited onto a cleaned cryogenically cold gold substrate. The same gas flow rate and depositing time ensure the same thickness of the deposited films. (**B**) Each freshly deposited film is bombarded by an electron beam at a given energy (ε). (**C**) The substrate is slowly heated, and the desorbed neutral species is detected according to a specific mass-to-charge ratio by a quadrupole mass spectrometer, providing a Temperature-Programed Desorption spectrum.

**Figure 2:**
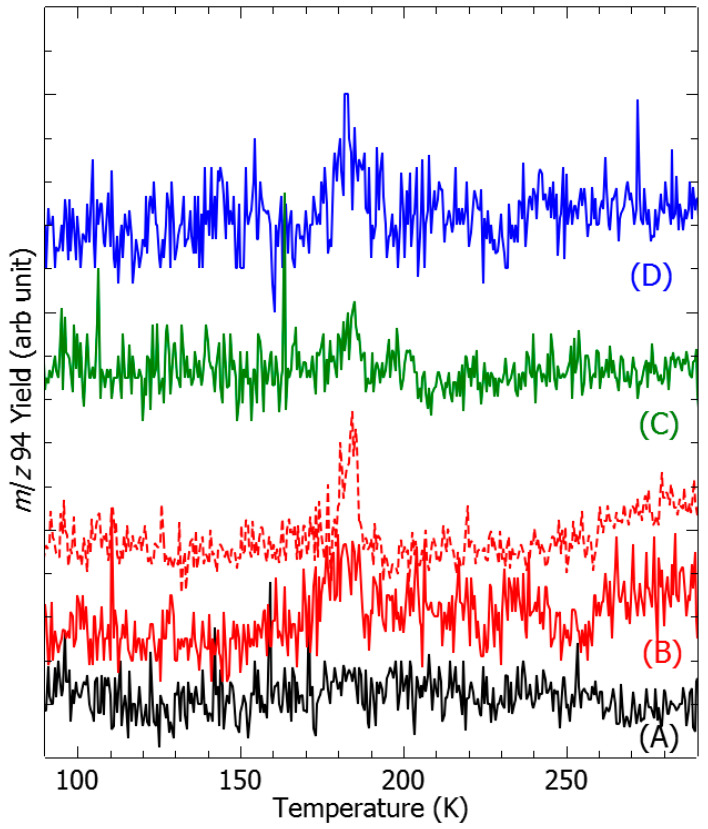
Temperature-Programmed Desorption spectra of phenol: ((A), black) no irradiation, and irradiation of 5 ML freshly deposited films by electrons of ((B), red) 0.8 eV 7.75 nA, ((C), green) 7 eV 28.7 nA, and ((D), blue) 9 eV 35.75 nA. The dashed red line represents the irradiation of ~10 ML thick fresh film by a 0.8 eV and 0.90 nA electron beam. The spectra are shifted for clarity.

**Figure 3:**
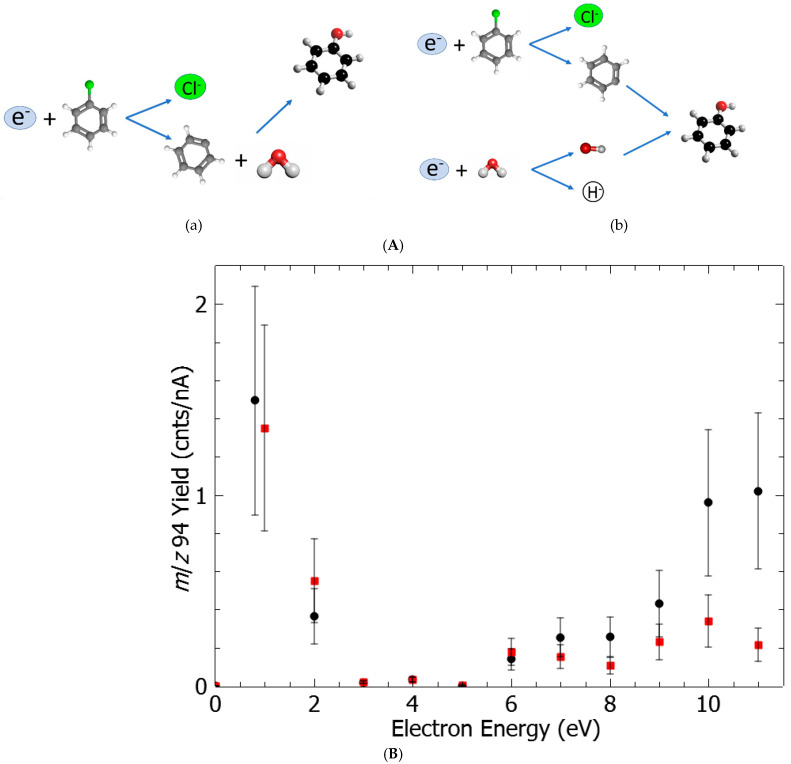
(**A**) Reaction (a) controls the production of phenol molecule below 2 eV, while both reactions (a) and (b) are likely to arise at electron energies above 4 eV. (**B**) The integrated yield and the yield normalized to the electron current are plotted as a function of the electron energy. The error bars were evaluated to be 40% from the average of 3 to 6 freshly irradiated films at the same energy. The electron energy resolution is 0.5 eV.

The principle of this experimental demonstration is depicted in Figure 1: (A) a vapor admixture of chlorobenzene/water is condensed onto a cold gold surface to form a molecular film; (B) the film is irradiated by electrons at a specific energy; and (C) the metal substrate is heated, and desorbed molecules are detected by means of mass spectrometry. In our experiments, after electron bombardment of a freshly deposited C_6_H_5_Cl:H_2_O film, new species were detected by mass-spectrometry at *m*/*z* 94 (Figure 2) and *m*/*z* 78 (SI1), which can be unambiguously assigned to phenol and benzene, respectively. It is to be noted that the spectra recorded at neither *m*/*z* 94 (Figure 2A) nor *m*/*z* 78 of the freshy deposited admixture without electron irradiation exhibit any particular structure and represent only a background signal. In contrast, spectra recorded after irradiation of the freshly deposited films at various electron energies show a broad desorption peak for phenol (*m*/*z* 94), peaking at a temperature of 182 K, similarly to benzene (SI1). The desorption peaks of phenol and benzene were integrated and normalized by the measured incident electron current prior to being plotted as a function of electron energy (Figure 3). Both phenol (black circles) and benzene (red squares) yield functions exhibit a peak at around 1 eV and structures at electron energies above 6 eV (Figure 3).

The interaction of low-energy electrons with chlorobenzene [11,12,13,14,15] and water [16,17] has been extensively studied separately in both the gas phase and condensed phase (i.e., molecular films). Dissociation of precursor molecules involves an energy-resonant process, known as dissociative electron attachment [9,10,18], representing the first step in the capture of the colliding electron in a molecular state to form a transitory anion. The latter undergoes dissociation to concomitantly produce a negative fragment ion and at least one neutral counterpart [9,10,18]. The studies of chlorobenzene [11,12,13,14,15] and water [16,17] show that at electron energies above 5 eV, the molecules dissociate into a chlorine anion, Cl^−^ (associated with a phenyl radical, •C_6_H_5_), and a H^−^ anion (associated with a hydroxyl radical, HO•), respectively, as dominant fragments. The yield function of the H^−^ anion exhibits resonant features at 7 eV, 9 eV, and 12 eV [16,17], while broad structures centered at approximately 6 and 9 eV have been reported for the Cl^−^ anion [11,14]. Thus, the generated associated phenyl and hydroxyl radicals may further recombine (e.g., HO• + •C_6_H_5_) to form phenol (Figure 3, reaction B). Such a recombination process may release a significant amount of energy [19], for instance, by generating UV photons, not detectable by the present set-up. Other products resulting from reactions induced by water-generated hydroxyl radicals, such as the formation of hydrogen peroxide via •OH + •OH → H_2_O_2_ (*m*/*z* 34), were observed but are not discussed here, as the mechanisms responsible for their formation have been thoroughly described elsewhere [20]. Reactions of O^−^ anions, also produced from the fragmentation of H_2_O, with aromatic compounds are known to arise via different pathways (e.g., hydrogen abstraction or substitution) [21]. In principle, such processes may also occur with chlorobenzene. However, since the cross-section of O^−^ anions is 10 to 15 times lower than that of H^−^ anions, and taking into account the rate of phenol production (Figure 2 and Figure 3), potential neutral species resulting from O^−^ anion reactions were not detected in the present work.

At electron energies below 2 eV, the yield of phenol resembles that of the Cl^−^ anion produced via DEA to chlorobenzene [11,12,13,14] and must arise from the fragmentation of this sole reagent, since no colliding particles, i.e., photons [22,23] or electrons [16,17], can fragment water molecules at such low energies. Indeed, gas phase experiments [11,12] combined with DFT calculations [13,14] have indicated that the 1 eV resonance transitory state leads to fragmentation of chlorobenzene for Cl^−^ anions and reactive phenyl radicals. Finally, the dissociation of chlorobenzene via C-Cl bond breakage at 1 eV can be supported by the reaction enthalpy [18], ΔH = D(C-Cl) − EA(Cl), with D(C-Cl) representing the bond dissociation energy (4.2 eV [24]) and EA(Cl) the electron affinity of chlorine atoms (3.61 eV [25]). From the known values, the reaction requires 0.6 eV, already accessible by the 1 eV electron (Figure 3, reaction A). On the contrary, the reaction enthalpy for water dissociation (i.e., D(O-H) = 5.15 eV [23] and EA(H) = 0.75 eV [24]) is not accessible at energies below 4.4 eV but only at higher energies (Figure 3, reaction B). Thus, the detected phenol and benzene can only result from reactions induced by the formed phenyl radical with the surrounding water molecules (Figure 3, reaction A). The dynamics of this reaction has been previously demonstrated [26]: the phenyl radical abstracts a H atom from H_2_O, forming benzene and •OH radicals, which in turn undergo a reaction forming phenol and •H radicals. The present observations of phenol and benzene formation are consistent with previous work on low-energy electron irradiation of benzonitrile/water films [27]. It is to be noted that this process may also arise at energies above 6 eV, in addition to the previously suggested direct radical–radical recombination process.

The production of phenol from electron irradiation of C_6_H_5_Cl:H_2_O films can be quantified relative to the amount of deposited ClBz. The production rates of phenol and benzene are estimated to be 43% and 63% in the 0–3 eV energy range and 5.7% and 37% in the 5–11 eV energy range (Figure 3). These rates can also be expressed relative to the deposited chlorobenzene molecules. The number of collected ionized molecules, N_i_, can be expressed as a function of ε, N_neut_, σ_i_, Ne, and L, which correspond, respectively, to the detection efficiency, number of neutral molecules, ionization cross-section, density of colliding electrons, and collision path length in the mass spectrometer: N_i_ = ε·N_neut_·σ_i_·N_e_·L. Assuming the detection efficiency is similar for chlorobenzene, phenol, and benzene, the number of new species generated per deposited chlorobenzene target, N_i_/N_i_^ClBz^, can be expressed as N_neut_/N_neut_^ClBz^·σ_i_/σ_i_^ClBz^, for which N_i_ and N_i_^ClBz^ and the respective ionization cross-sections are as shown in Figure 3, (SI2) and Ref. [28]. Thus, N_neut_/N_neut_^ClBz^ is estimated to be approximately 2.15% and 1.17% for the production of phenol and benzene, respectively, under the present experimental conditions, which involve electron irradiation with a current of µA over an area of 0.3 cm^2^ and a film composition of 25% chlorobenzene and 75% water.

In general, chemical reactions taking place in a medium involve a large ensemble of molecules, and statistical observations can obscure the dynamics of individual molecules. From this work, two main conclusions can be drawn. The present method demonstrates the ability to selectively target specific molecules that are randomly spatially localized in films with few mono-layers to achieve a reaction; this has also been verified for films of ~10 MLs. This is feasible by controlling the energy of the irradiating electrons, leading to two distinct reaction pathways: (1) association of two radicals, phenyl and hydroxyl (Figure 3, reaction B), at electron energies above 6 eV, or (2) initiation of secondary reactions by phenyl radicals at energies below 2 eV (Figure 3, reaction A). It is to be noted here that, in terms of comparative UV photon vs. electron efficiencies, it is very likely that the latter surpass photons. Both photons and electrons induce C-Cl bond cleavage, as the first step in subsequent chemical processes. It is important to note that while the decomposition cross-section of chlorobenzene on Ag induced by photons has been estimated to be 3 × 10^−21^ cm^2^ (at a wavelength of 254 nm), and decreases with increasing wavelength [15], that induced by electrons has been measured to be three orders of magnitude higher, i.e., 1.4 × 10^−17^ cm^2^, at an energy of 0.85 eV [11].

## 3. Material and Methods

The experimental UHV (5 × 10^−10^ mbar) set-up has been described elsewhere [5]. The chlorobenzene (ClBz) and water (spectroscopic-grade, SIGMA-Aldrich, St. Louis, MO, USA) are degassed by repeated freeze–pump–thaw cycles under vacuum before use. At room temperature, ClBz (3 mb; ~10%) and H_2_O (28 mbar; ~90%) vapors are pre-mixed in a 27 mL mixing cell. The admixture is injected for condensation onto an 90 K cryogenically cooled Au substrate. This substrate is resistively heated (>450 K) for cleaning, with the chamber is free from contamination (S3), prior to each deposition, and the temperature is measured (±2.5 K) using an E-type thermocouple fixed onto the gold substrate. The volumetrically calibrated effusing gas quantity (MKS Baratron type 127) provides an estimate of the thickness of the film, which is estimated to be typically 5 monolayers (MLs), ±40%. It is noteworthy that, in these experiments, knowledge of the exact thickness of the film is not necessary, but the film must be sufficiently thick to avoid the possible effects of the metal substrate (i.e., quenching or enhancing process) and thin enough to minimize multiple-electron inelastic scattering [29], which would modify the energy of the reacting electrons. The monoenergetic electron beam (ΔE~250 meV, a few tenths of nA, ~0.3 cm^2^), provided by a trochoidal electron monochromator, impinges normally on all deposited films for 50 min. The electron energy is calibrated by fast recording of the onset of the electron transmission curve [30], and rescaled every 30 s until no appreciable shift is observed after the charging of the film. Otherwise, the film would be irradiated at different electron energies, and thus, the fragmentation process would consequently be modified. After film irradiation, the substrate is heated at a rate of 12 K/min for the thermal desorption. The desorbed neutral species are detected by recording the associated positive ions produced from the 70 eV electron ionization of the molecules inside the quadrupole mass spectrometer (QMS, Balzer). Therefore, at a given electron irradiation energy, the yield of the neutral species as a function of the substrate heating temperature provides the temperature desorption spectrum (TDS). At each electron energy, measurements are repeated in a standard manner 3 to 6 times, providing a reproducibility value of ~40%.

## 4. Conclusions

This study demonstrates that low-energy electrons (<10 eV) can be used to selectively initiate chemical reactions in heterogeneous molecular films by targeting specific components through resonant dissociative electron attachment (DEA). Using a prototypical model system of chlorobenzene (C_6_H_5_Cl) in water ice, it is shown that electrons with energies below 2 eV selectively fragment chlorobenzene, producing Cl^−^ and phenyl radicals, which subsequently react with surrounding water molecules to yield phenol and benzene. Through irradiation of the chlorobenzene/water ice mixture, it is shown that electrons with energies below 2 eV selectively fragment chlorobenzene, producing Cl^−^ and associated phenyl radicals that react with water to form phenol and benzene. These reactions are energy-dependent and highly selective, with no water dissociation occurring at these low energies. At higher electron energies (>6 eV), multiple reaction pathways open, involving radicals from all components and leading to a broader product distribution.

Targeting a specific compound provides an analytical tool for unraveling, with unprecedent detail, the reaction dynamics of individual molecules in a medium. Furthermore, the information gained from an electron-induced reaction can potentially contribute to roboticized AI-assisted/guided chemistry, in a manner similar to that used in photocatalytic synthesis [31]. This approach could potentially be applied in both research and practical applications, such as STM-induced synthesis [6,7,8], surface plasmon resonance-driven synthesis [32], or electron-induced catalysis [33].

## Data Availability

The data are contained within the article and Supplementary Materials.

## Supplementary Materials

The following supporting information can be downloaded at: https://www.mdpi.com/article/10.3390/ijms26178751/s1.
