# Supplementary material for: Activation of Specific Reagents in Molecular Films by Sub-Ionization Electrons: Chlorobenzene/Water Films
Source: Int J Mol Sci. 2025 Sep 8;26(17):8751. doi: 10.3390/ijms26178751 (PMC12429294; doi:10.3390/ijms26178751)

Activation of Specific Reagents in Molecular Films by Sub-ionization Electrons: Chlorobenzene:water films

Hassan Abdoul-Carime[1], Janina Kopyra[2]

[1]*Universite Claude Bernard Lyon 1, Institut de Physique des 2 Infinis, CNRS/IN2P3, UMR5822, F-69003 Lyon, France*

[2]*Faculty of Sciences, Siedlce University, 3 Maja 54, 08-110 Siedlce, Poland*

Corresponding author: hcarime@ipnl.in2p3.fr

S1. Temperature Desorption Spectrum of m/z 78 species (Benzene). Note that this recorded spectrum is further temperature rescaled accordingly, providing a temperature desorption peak of m/z 78 at 180 K

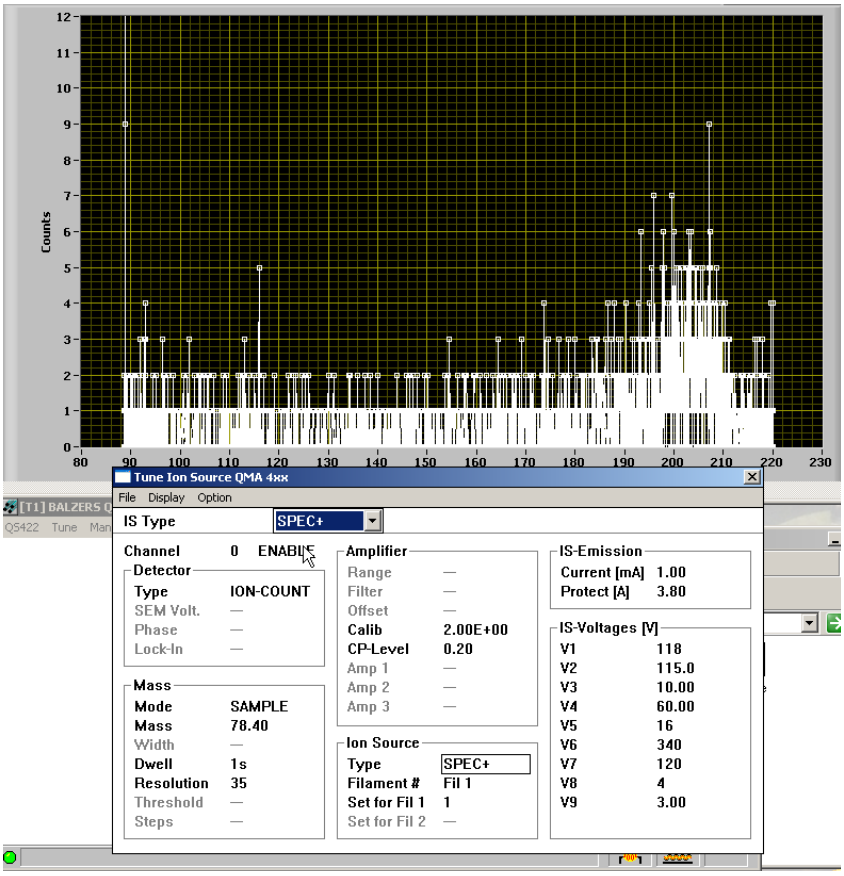

S2. Temperature Desorption Spectrum of Chlorobenzene from ~ 5ML film of Chlorobenzene:water (10%:90%). The yield presents peaks at T = 164 K, 178 K with a shoulder at 232 K. The first temperature can be correlated with the TPS signal of desorption of a pure water film from the gold film, indicating that chlorobenzene is expelled with water molecules, while the two latter temperatures may be related to the desorption of multilayer and monolayer of chlorobenzene, respectively, from the Ag(111) film.

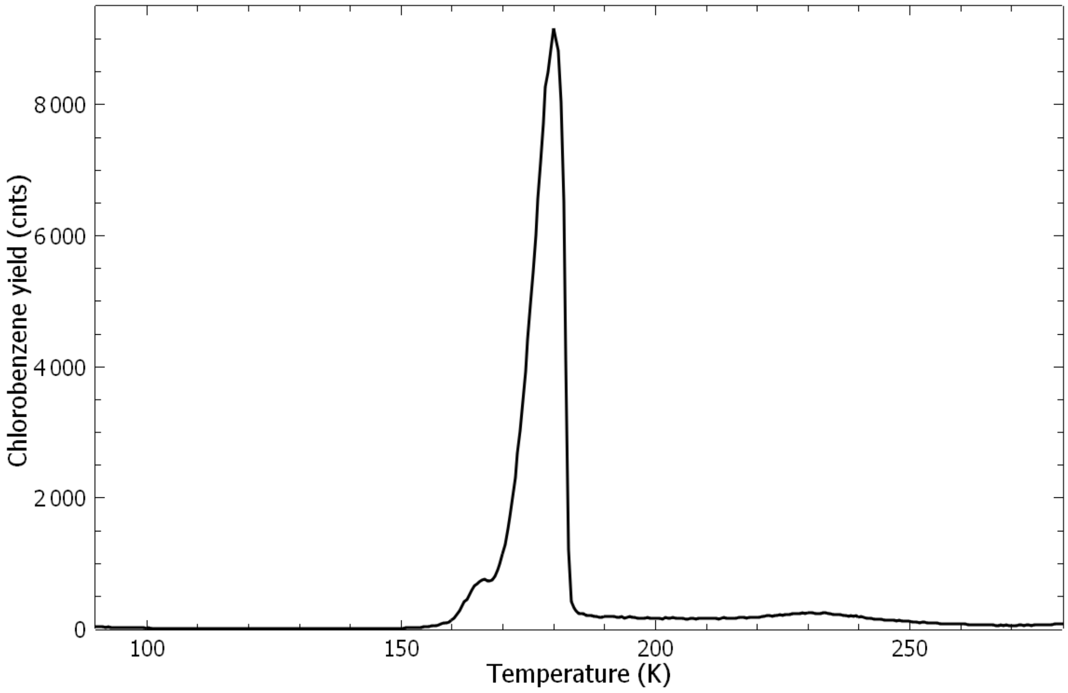

S3. Background gas : Background gas is free from contaminant (e.g., m/z 80-150 region) to ensure that the detected m/z 94 (associate to phenol) after electron irradiation has not been brought initially by contamination.

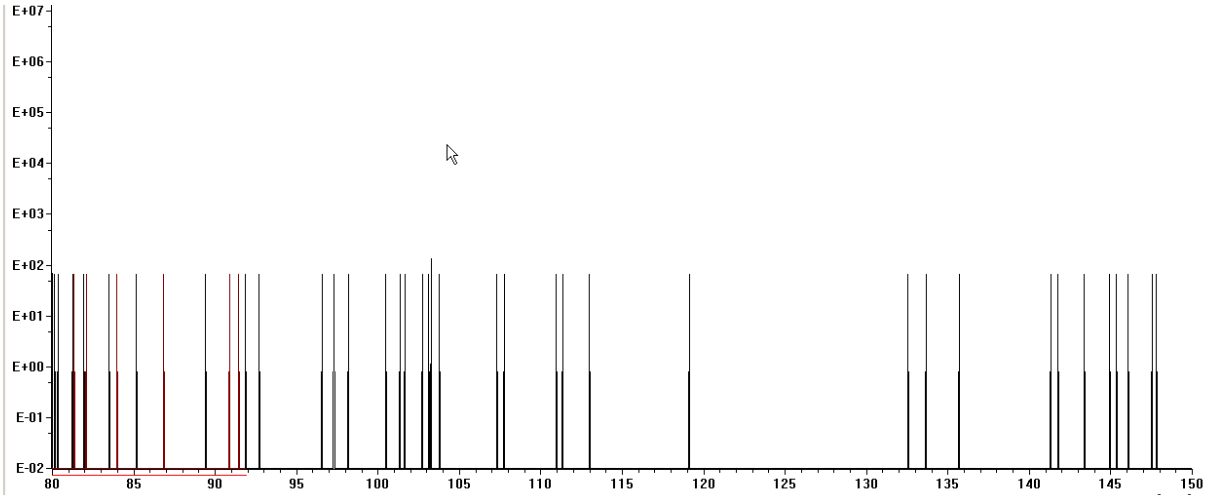

Supplement: Supplementary file 1 [file ijms-26-08751-s001.zip › ijms-3788896-supplementary.pdf]
